# Improved MAC layer protocol of Wifi for satellite network

**DOI:** 10.1371/journal.pone.0221551

**Published:** 2019-09-06

**Authors:** Sili Liu, Jianyun Chen, Chao Ma, Lingxiao Zhu

**Affiliations:** College of Intelligence Science and Technology, National University of Defense Technology, Changsha, China; Chongqing University of Posts and Telecommunications, CHINA

## Abstract

With the development and universal application of satellite technology, an important way to expand the function of satellites is setting up inter-satellite networks to make them work together. Traditional satellite networking methods generally adopt a fixed time slot allocation method, which is not suitable for small satellite groups with low latency and high throughput requirements. In order to solve this problem, it has been proposed to apply the traditional Wifi protocol in satellite networking. As there are differences between satellite networks and terrestrial networks, it’s necessary to improve the traditional 802.11 protocol. The Media Access Control (MAC) protocol in 802.11 is improved in this paper, which mainly includes the adaptive algorithm of maximum contention window size and the growth algorithm of Contention Window (CW) size. The maximum contention window is adjusted according to the conflict state of the current network, which makes the network accommodate more satellite nodes. The CW growth algorithm improves the traditional Binary Exponential Back-off (BEB) algorithm, where the CW is designed according to the priority of the data frame or the network load. In this way, high-priority satellite accusation information will have higher reliability or tolerate greater network load.

## Introduction

In recent years, space technology has developed rapidly. As an important platform for space communication, satellites have increasingly higher requirements for performance, power consumption, volume and reliability [1]. In order to accomplish a specific task, it is generally required that a plurality of satellites form a constellation to communicate with each other and work together. In the field of satellites, the research of wireless networks has become a research hotspot with the development of aerospace technology and the growth of its business needs. Many countries have made full use of space resources to deploy a large number of spacecraft including low- orbiting and medium-orbiting satellites, small satellites, drones and the airships, to obtain spatial information in a timely manner [2]. With increasing number of spacecraft nodes, how to organize a network within them to achieve efficient message transmission has become the main research direction of current space networks [3, 4].

In the satellite networking protocol, an important content is the media access control protocol, which is used to avoid the situation where multiple signals are transmitted at the same time or data packets are lost [5]. As the satellite has a fixed orbit and its position coordinates and motion state can be predicted according to the ephemeris for a period of time, the existing satellite network MAC protocol is mainly based on the pre-planned MAC layer protocol [6]. The advantage is that the reliability is high, and each node has the opportunity to occupy the media in a period of time; the disadvantage is that when no node sends a message, it will occupy a media for a period of time, which will waste resources and increase network delay.

The selection of satellite networking protocol is closely related to the network topology of satellite constellation. Generally speaking, for the constellation networking with medium and high orbit, the relative positions between the constellations change periodically owning to the long communication distance. In this connection, the network is usually established by planning routing and channel slots in advance. In the small satellite group in low-orbit, the distance between satellites is dozens to hundreds, and the relative position relationship between satellites changes frequently. The number of satellites is generally 3 to 8, which is mainly used for space environment exploration, or for internal networking of a cluster of subnets in a large rapid reconnaissance response constellation.

With the increasing demand for satellite functions, higher requirements for the real-time and flexibility of satellite networks is needed. In order to solve this problem, the media access control method adopted by the inter-satellite network is generally based on CSMA/CA protocol [7–9]. The existing MAC layer protocol uses the BEB algorithm in 802.11 as the mainstream algorithm (described in Appendix) [10, 11].

The problems faced by the satellite network MAC layer protocol can be summarized as follows:

1. The distance between nodes in the terrestrial network is generally within 100 kilometers, and the propagation delay is generally several nanoseconds and microseconds. However, for satellite networks, the distance between nodes is generally several hundred thousand kilometers, and the propagation delay can reach several milliseconds [12]. Therefore, the inter-frame time interval and other parameters defined in the terrestrial network needs to be reconsidered.
2. In the actual network, the information and business we transmit on the satellite network are different, and the reliability and real-time requirements for the data packets are different. For some of the more important allegations, it needs to be reliably delivered to the destination satellite in a short period of time; while some data are transmitted, the delay requirement and reliability requirement are relatively low [13].

For the above two problems, many people have already given some solutions. Especially for the problem of the propagation delay, the solution commonly adopted is to make slot time planning according to the propagation delay of the inter-satellite link [14, 15].

In 2008, Tanya Vladimirova and Kawsu Sidibeh proposed an adaptive improvement method for the IEEE 802.11 protocol for satellite network applications [16]. In the same year, the Space Center at the University of Surrey used the Cube-Sat platform to verify the IEEE 802.11 protocol after the time parameter modifying according to the satellite transmission distance [7–9]. Further improvements were made in ViaSat-1 satellites in 2011 and high-throughput satellites such as HughesNet’s Jupiter in 2012, which increases the downlink data rate from 1–3 Mbit/s to 12–15 Mbit/s or higher [17]. In 2013, Shen Zeshu of Aerospace Dongfanghong Satellite Co. Ltd. simulated the applicability of 802.11 in inter-satellite links. In 2016, Liu Shuo of Beijing University of posts and telecommunications proposed a rate-based optimal relay selection MAC. Agreement [18]. In order to reduce the media waste caused by the propagation delay, an improved scheme of transmitting and receiving two nodes simultaneously at the same slot time has been proposed in 2017 [19]. These protocols have carried out on-board simulation verification of the ground networking protocol, which provides the topic support and theoretical basis for our research work. The above improved MAC layer protocol still uses the BEB algorithm. This paper mainly aims to reduce the delay, increase the probability of successful transmission of high-priority frames and increase the network load that the protocol can bear by improving the back-off collision avoidance algorithm.

## Materials and methods

We improve the MAC layer protocol in 802.11 to adapt to the characteristics of the satellite network. The main improvements focus on the following three aspects:

1. The parameters including the inter frame time interval and slot time of the MAC layer protocol are adjusted according to the inter-satellite transmission distance.
2. In network transmission, the network working conditions are different in different time periods, leading to its load or throughput to change with time. These factors will affect the quality of the MAC layer protocol. Therefore, we improve its MAC layer protocol, which adaptively adjusts its maximum contention window size according to the network collision situation.
3. Optimize the back-off time growth algorithm to make it more suitable for the different transmission requirements of various priority frames and different network load.

### Modify time parameter

When using the terrestrial wireless network protocol for satellite networks, we need to modify the parameter values related to the propagation delay to ensure the normal operation of the protocol. In various frame intervals of 802.11b, the Distributed Coordination Function IFS (DIFS), time of slot (Slot), and acknowledgement time (Ack) are related to air propagation delay. When the distance between nodes increases, the propagation delay increases. The parameters need to be redefined. The simplest and most effective way is to correct the delay parameters according to the maximum value of the actual inter-satellite link length, so that the MAC layer protocol applicable to the satellite network can be obtained.

In CSMA/CA, the time of slot contains three aspects, and its calculation formula is as (1) [20]:

Slot=MDT+TRT+APT (1)

Where Slot means the time of slot, MDT means the media detection time, TRT means transceiver antenna conversion time and APT means air propagation delay time. Among them, APT is related to the communication distance, and its calculation formula is as (2) [20]:

APT=2×Dist/c (2)

Where Dist is the maximum communication distance, and c is the speed of light. In an inter-satellite network, APT is much larger than MDT and TRT.

DIFS is the shortest media idle time in a competing service. If the media is idle longer than DIFS, the satellite can access the media immediately, and its calculation formula is as (3) [20]:

DIFS=SIFS+2×Slot (3)

When applied to the inter-satellite network, Slot will be changed, which is calculated according to Eq (1).

Ack: The time at which the node waits for the receiver satellite to reply to the response frame. If the time is exceeded, it is determined that the transmitted frame is lost. It can be calculated as follows (4) [20]:

Ack=FTT+2APT+SIFS+ATT (4)

Where FTT means the frame transmission time, ATT means acknowledgement transmission time. The parameters in Eq (4) are constant except APT, while APT can be calculated according to Eq (2).

### Adaptive maximum contention window

According to the principle of BEB algorithm, its performance is affected by the value of Maximum Contention Window (CWmax). When there are many satellite nodes in the network, if the set CWmax is relatively small, the probability of collision of nodes is relatively large. After the conflict occurs, the node will be forced to double the size of its contention window, which will result in inefficiency. On the other hand, if the load on the network is light and the competition is weak, even the Minimum Contention Window (CWmin) may cause the node to wait for transmission for too long. Under normal circumstances, the number of satellite nodes in the satellite network is fixed, so there will be no nodes entering the network or leaving the network as often as the terrestrial network, that is, the network situation will not change greatly. This feature of satellite networks allows us to reasonably set the maximum contention window based on the number of satellite nodes, the collision situation and the amount of data to be sent in the network. And we can set a relatively small value for CWmin.

The value of CWmax is related to the current network state. The algorithm we proposed modifies the size of CWmax in real-time: Feedback information about the media condition will be added to the BEB algorithm to calculate the size of CWmax. The optimized CWmax is related to the number of nodes in the network, the number of nodes colliding at the moment, and the amount of data of each node to be sent. The calculation formula is as (5):

$$CW_{\text{max}}=NS\times \sqrt{n}\times T_{c}\times Slot$$ (5)

Where NS is the number of nodes that collide at the moment, n is the number of satellite nodes in the network, Tc is the collision time, and SlotTime is the value of slot time.

### Contention window size growth algorithm

When performing media access, if the media is busy at this time, the back-off time needs to be recalculated, and in the traditional 802.11 back-off time is a random number not greater than CW which is binary exponential growth. Considering that the frames transmitted by the satellite network include different types of data frames and control frames, the required network performance is also different in various frames. For example, control frames often require higher reliability and lower latency characteristics, so that allegation information can be processed in a timely manner, and the network can update the current parameters of the maintenance network in real time: Data frames allow for lower real-time and reliability. For different types of messages and business types, we give them different priorities and design them separately when designing the CW growth algorithm. At the same time, the CW can be adaptively changed according to the degree of network conflict, thereby increasing the tolerance of the algorithm to the network load situation.

According to above discussion, the BEB algorithm can be improved, and the calculating formula of CW is as (6):

CW=(F×NBDT+max(F−1,0)×DTET)×Slot (6)

Where NBDT and DTET are related to the hardware parameters of the device. NBDT is the Net Busy Detect Time and DTET is the time required for the DTE or modem to stop listening for received data or squelch detect and to activate the radio’s push-to-talk. In the simulation below, NBDT is 8ms and DTETURN is 40ms. The value of CW is mainly determined by the F value. The F value calculation is explained below, and three calculation methods are given.

#### 2.3.1 Random-CW

Random-CW ensures that each satellite node has equal access opportunity to the network. The stochastic feature also provides a solution if an access violation occurs. That is to say a new CW will be given for next time media access. Different CW values will be used each time when nodes try to occupy the media. Firstly, in order to slow down the growth of the back-off time, we let F increase evenly. And the formula for calculating F is (7):

F=Integer((C+1)×0.75×NS)+C) (7)

As shown in (7), NS is the number of nodes participating in the competition at this time, and C is the number of collision times of the node to send this frame. The F value should be an integer rounded up between 0 and (3/4) NS, and the F value increases every time the data frame collides by ((3/4)×NS +1).

#### 2.3.2 Priority-CW

Priority-CW algorithm is improved on the basis of random access, in which F increases evenly. The main improvement is reflected in the frame priority (MP). The value of F in Priority-CW is calculated as (8):

F=MP×(C+1) (8)

Where the frame priority MP is calculated as (9):

$$MP=\{\begin{array}{c} (NS+1)\times C,high-priority\\ 2\times (NS+1)\times C,low-priority \end{array}$$ (9)

Where NS is the number of nodes participating in the competition at this time. When the frame priority is high-priority, the MP is (NS+1) ×C, and when the frame priority is low-priority, the MP is (2×C×(NS+1)).

At the same time, the CWmax of the high-priority frame is set to K1 times of the low-priority frame, and the maximum collision times of high-priority frame is set to the K2 times of the low-priority. The values of K1 and K2 are optimized according to the specific network simulation results. The purpose of this is to ensure that the high-priority frame gets a higher probability of successful transmission when the delay meets the space network transmission requirements.

#### 2.3.3 Load-CW

Load-CW algorithm takes into account factors such as network expected load and frame priority. While ensuring the transmission delay of various priority frames can meet the requirement. It also guarantees that the network can tolerate a greater load. Each priority level has a pseudo-random F value that is determined by the number of satellite nodes in the network, the network percentage of a particular priority frame, and the expected traffic load. The calculation formula of urgent and priority frame is as (10):

F=Integer(MIN+RN×(MAX−MIN)+C) (10)

The calculation formula of routine frame is as (11):

F=Integer(MIN+RN×(MAX−MIN)×(C+1)) (11)

Where RN is pseudo-random number in the range 0.0 to 1.0. MAX and MIN are integer values. For messages of different priorities, the calculation rules are as follows:

For urgent frames:

$$\{\begin{array}{c} U\_MIN=0\\ U\_MAX=USIZE+1 \end{array}$$ (12)

For priority frames:

$$\{\begin{array}{c} P\_MIN=U\_MAX+1\\ P\_MAX=P\_MIN+PSIZE+1 \end{array}$$ (13)

For routine frames:

$$\{\begin{array}{c} R\_MIN=P\_MAX+1\\ R\_MAX=R\_MIN+RSIZE+1 \end{array}$$ (14)

Where USIZE is the additional random number generated for the urgent frame, PSIZE is the additional random number generated for the priority frame, RSIZE is the additional random number generated for the routine frame, and the minimum range size between MIN/MAX is 2.

Additional random numbers of different priorities are integers calculated based on the percentage of particular priority frames and (ADJ_NS) values, as shown in (15):

$$\{\begin{array}{c} USIZE=U\times ADJ\_NodeNumber\\ \begin{array}{l} PSIZE=P\times ADJ\_NodeNumber\\ RSIZE=R\times ADJ\_NodeNumber \end{array} \end{array}$$ (15)

The additional random numbers of the above three priorities are rounded to the nearest integer. U is the percentage of urgent frames expected to be transmitted in the network, P is the percentage of the priority frames expected to be transmitted in the network, and R is the percentage of routine frames expected to be transmitted in the network. The ADJ_NodeNumber is determined by the number of nodes in the satellite network and the network expected load. The calculation formula is as (16):

ADJ_NodeNumber=Integer(n×TL) (16)

Where n is the number of satellite nodes in the space network and TL is the expected traffic load factor, which is 0.5 for large traffic load, 1.0 for normal traffic load, and 1.5 for a light traffic load.

## Results

In this part, we simulated the improved back-off collision avoidance algorithm and perform horizontal and vertical comparison. Firstly, we analyzed whether the parameters in the algorithm which improved parameters can be adapted to the space network transmission requirements. Secondly, we analyzed the impact of the adaptive CWmax on the performance of the algorithm. Finally, three back-off collision avoidance time algorithms proposed above: Random-CW, Priority-CW, Load-CW are simulated, and the main advantages of Priority-CW and Load-CW are analyzed.

### Applicability analysis

The back-off collision avoidance algorithm of the 802.11 BEB mechanism is verified that it’s applicable to the satellite network. The improved methods proposed by us are mainly applicable to subnets of small star clusters with the number of satellites ranging from 3 to 8 and the communication distance ranging from tens to hundreds of kilometers. The antenna communication rate of the satellite is limited by volume, weight and other factors, which are different greatly among satellites, and the value is 4Mbps in simulation. Maximum number of retransmissions is 7 according to the Eq (17). The input conditions are as Table 1:

**Table 1:** The put parameters in simulation.

Parameters	Value
the number of satellite nodes	4
maximum communication distance	600km
maximum number of collisions	12
Data transfer rate	4Mbps
maximum number of retransmissions	7
Simulation time	>20minutes
message priority ratio(Priority-CW)	allegation frame30%, data frame70%
frame priority ratio (Load-CW)	urgent frame 25%, priority frame 25%, routine frame 50%

After changing the slot parameters of 802.11 according to the previous calculation scheme, the simulation results are as follows.

As shown in S1 Fig, the delay of all the frames in the network is less than 3 seconds. After simulation calculation, the average delay in simulation time is 0.0722s. The maximum delay is 2.796s, which can basically meet the needs of the satellite network.

S2 Fig shows the probability that a frame can be successfully received by the destination node without retransmission. The small picture in this S2 Fig is a magnified part of the simulation in the beginning of simulation. It can be seen that as the simulation time becomes longer, the probability of successful frame transmission becomes more stable. After stabilization, the successful transmission rate (p) of the frame is 88.62%. When we set the maximum number of retransmissions to 7, the probability of frame failure transmission is P, which is calculated as Eq (17):

$$P={\left(1-p\right)}^{7}=2.\text{4717}\times 10^{-7}$$ (17)

In S3 Fig, the red curve shows the throughput of the network during the simulation, which includes the amount of data that was sent successfully or failed and can be used to reflect the current load on the network. And the blue curve shows the effective throughput, which only includes the amount of data that was sent successfully.

According to the statistical results, the network throughput during the simulation time is 1187.3 kbps and the effective throughput is 1051.9 kbps. From S3 Fig, it can be seen that the throughput is gradually stabilized as the simulation time increases.

### Improvement of the CWmax

The adaptation of the CWmax mainly increases the flexibility of the network protocol and reduces the delay to a certain extent. The supported nodes can have higher flexibility without changing other parameters. The basic performance parameters of the algorithm are as follows.

As S4 Fig shows, the delay of all the frames in the network is less than 1 seconds. After simulation calculation, the average delay in simulation time is 0.0652 s. The maximum delay is 0.672s, which can basically meet the needs of the satellite network.

S5 Fig shows the probability that a frame can be successfully received by the destination node without retransmission. The small picture in this S5 Fig is a magnified part of the simulation in the beginning of simulation. As S5 Fig shows, after stabilization, the successful transmission rate (p) of the frame is 88.83%.

S6 Fig shows the network throughput during the simulation, and the stable network throughput is 1187.5kbps. S6 Fig shows the network effective throughput during the simulation, and the stable network throughput is 1055.1kbps. Compared with the non-adaptive maximum contention window, the average and maximum delay is reduced, while the network throughput, effective throughput and the successful transmission rate of the frame are almost unchanged. The performances of this algorithm basically satisfy the requirements of satellite transmission.

It can be inferred from the principle of its adaptation that the advantages of the algorithm mainly reflect the greater tolerance of the network to the change in the number of satellite nodes. After the introduction of the adaptive algorithm in the maximum contention window, the comparison between the basic performance of the network and that before the introduction of the adaptive algorithm is shown in the Table 2:

**Table 2:** Performance comparison before and after introduction adaptive algotithm.

Parameters	BEB	Improved the CWmax
Average delay/s	0.0722	0.0652
Maximum delay/s	2.796	0.672
Probability of frame successful transmission	88.62%	88.83%
Throughput/kbps	1187.3	1187.5
Effective throughput/kbps	1051.9	1055.1

It can be seen from Table 2 that the introduction of adaptive maximum contention window can reduce the maximum delay, thus reducing the delay jitter. It can be inferred from the principle of its adaptation that the advantages of the algorithm mainly reflect the greater tolerance of the network to the change in the number of satellite nodes. S7 and S8 Figs show the frame average delay, maximum delay, the successful transmission probability and effective network throughput of change over the number of nodes, respectively. The red curve is the result when the adaptive maximum contention window is not introduced and the green curve is the result after the introduction of the adaptive maximum contention window.

The maximum contending window can be adjusted according to the number of conflicting nodes in the network. When the number of nodes in the network is large, the load is large, and there are many collision nodes, the maximum contention window size is increased. When there are few nodes in the network, the load is small, and the collision node is small, the size of the maximum contention window should be reduced. In other words, the size of the maximum competitive window is positively correlated with the number of network nodes. Therefore, after the introduction of adaptive Windows, the number of nodes that can be accommodated in the network will increase.

It can be seen that as the number of network nodes increases, the average delay and maximum delay will gradually increase, and the probability of successful frame transmission will gradually decrease. When the curve is abrupt and average delay larger than 10s, the current parameter settings can no longer adapt to the current network situation. It can be seen from the above experimental results that after introducing the adaptive CWmax, the number of nodes that the network can support is larger without changing other parameters, that is, the flexibility of the network is greater. When a few new nodes join the network, the satellite network can be guaranteed to operate normally.

### Growth mode of the CW size

#### Random-CW

S9, S10 and S11 Figs are the simulation result of Random-CW algorithm. As shown in S9 Fig, it is a transmission delay diagram of each frame sent by four nodes. During the simulation time, the transmission delay of all frames does not exceed 1 s. Through statistical calculation, the average delay of frames in the network is 0.066s, and the maximum delay is 0.672s. S10 Fig shows the probability of successful transmission of frames without retransmission. After stabilization, the probability of successful transmission in the simulation time is 88.68%.

During the simulation time, the throughput of the network is shown in S11 Fig, where the abscissa represents the time slot and the ordinate represents the network throughput. We can see that the stable throughput is 1196.1 kbps. S11 Fig shows the effective throughput, and the stable value is 1060.2 kbps.

The reasons for the improvement of network performance are as follows: the transmission distance between satellites is relatively large, and the contention window adopts the exponential growth mode, which will cause the withdrawal time to increase rapidly Thus, it will lead to longer delay. The more uniform growth mode can ensure the stability of network delay and reduce the delay jitter.

#### Priority-CW

In the Priority-CW algorithm simulation, two message priorities are considered, where high priority indicates that the transmitted information is allegation information, and low priority indicates that the transmitted information is digital transmission information. The basic simulation results of the Priority-CW algorithm are as follows.

As S12 Fig hows, the delay of all frames is less than 2s. S13 Fig shows that the probability that a frame can be successfully received by the destination node without retransmission is larger than 90%. S14 Fig shows the network throughput and effective throughput during the simulation time, in which the red curve stands for throughput and the blue curve stands for effective throughput. The specific simulation results are shown in Table 3.

**Table 3:** The simulation result of Priority-CW algorithm.

Performance	Value
Average delay/s	0.0844
Maximum delay/s	1.216
Probability of frame successful transmission	0.9116
Throughput/kbps	1183.2
Effective throughput/kbps	1078.8
Average delay of allegation frame/s	0.1003
Average delay of data frame/s	0.0777
Maximum delay of allegation frame/s	1.216
Maximum delay of data frame/s	1.164
Probability of allegation frame successful transmission	0.9684
Probability of data frame successful transmission	0.8998

It can be seen from Table 3 that the average delay of the allegation information and the data information is basically the same, and both can meet the space network transmission requirements. However, the probability of successful transmission of the allegation information is much higher, which means the reliability of the allegation information transmission is higher than that of data information. When the requirement for the bit error rate is constant, the number of re-transmissions of the allegation information will be much less, thereby reducing the transmission layer delay caused by retransmission. The above results is in line with the original intention of algorithm design.

#### Load-CW

The basic simulation results of the Load-CW algorithm are as follows. S15, S16 and S17 Figs show the network delay, the probability of frame successful transmission, the network throughput and effective network throughput respectively.

As shown in S15 Fig, the transmission delay of all frames is no more than 0.5s. Through statistical calculation, the average delay of frames in the network is 0.0368 s, and the maximum delay is 0.452s. S16 Fig shows the probability of successful transmission of a frame without retransmission. The probability of success transmission in the simulation time is 93.76%. S17 Fig shows that the stable throughput is 1178.5kbps and the stable effective throughput is 1105kbps. Table 3 shows basic performance comparison of the above algorithms, where delay represents the average delay, delay-max means the maximum of delay, probability means the probability of successful transmission and throughout means the satellite network throughput. Throughput can reflect the load of the network, and effective throughput can reflect the amount of data successfully transmitted by the network. We can see from Table 4 that the performances such as delay and reliability of Load-CW are relatively better than the previous algorithms.

**Table 4:** Basic performance comparison of the above algorithms.

Algorithm	Delay/s	Delay-max/s	Probability/%	Effective Throughput/kbps
BEB	0.7220	2.796	88.62	1051.9
Adaptive CWmax	0.0652	0.672	88.83	1055.1
Random-CW	0.0660	0.672	88.68	1060.2
Priority-CW	0.0876	1.644	91.22	1078.8
Load-CW	0.0368	0.452	93.76	1105.0

The main advantage of Load-CW is that it can adjust its own contention window according to the network load. The flexibility is relatively strong, that is to say, the network load that can be tolerated is relatively large. As can be seen from the table above, this algorithm can improve the traffic load that the network can bear, and at the same traffic load, other performance is better. The figures below illustrate the comparison of the performance with different network load.

S18 and S19 Figs sequentially show the average delay, the maximum delay, probability of frame successful transmission, and the network throughput of change over the variation of network load. The red curve indicates the simulation result using the traditional BEB algorithm, and the blue curve indicates the simulation result using the Load-CW algorithm. When the delay curve is abrupt, it means that the parameters in the algorithm can’t meet the requirements of the current network load. From S19 Fig, we can see the maximum network throughput that the Load-CW algorithm and the traditional BEB algorithm can support in the current simulation environment. It can be seen from the comparison chart that the Load-CW algorithm has a large adaptability to the network load.

## Discussion

From the foregoing discussion and simulation analysis, the improved terrestrial wireless MAC layer protocol can be applied to the media access control in satellite networks. The main improvements focus on the following three areas. The protocol is improved in three aspects. Firstly, the parameters related to the propagation delay in its time series model are recalculated in order to adapt it to long-distance transmission in space. Additionally, the value of the CWmax is optimized according to the current network conflict situation in real-time. The effect of the improvement is that the average delay of the network is reduced, and the number of satellite nodes that satellite network can accommodate is increased. Finally, he BEB algorithm is improved. Due to the large time slot of the satellite network, the traditional binary growth mechanism has a great increase in back-off time, which will cause a large delay. That will cause the satellite network can’t adapt to large network load, and can’t meet the high reliability requirements of the satellite allegation information. In terms of the frame priority, the algorithm can be designed to optimize the high-priority reliability while ensuring that the allegation information delay is not too high; In terms of network load, the load factor can be added to the back-off time calculation, so that the algorithm can withstand greater network load without changing other parameters. Satellite networking is a hot issue in the current satellite field. The mature ground network protocol is an important support for studying this problem. However, to verify whether the related protocols in wireless Wifi technology, wireless sensor networks, Mesh networks, and Ad hoc networks can be better applied in space-based networks, there are still many improvements and verification work that need to be done.

## Supporting information

S1 Dataset Raw date. This document contains a link to get the raw data.
(DOCX)

S1 Fig The delay of each frame sent by the four nodes. (TIF)

S2 Fig The probability that a frame can be successfully received by the destination node without retransmission. (TIF)

S3 Fig The throughput and effective throughput of the network during the simulation. (TIF)

S4 Fig The delay of each frame sent by the four nodes. (TIF)

S5 Fig The probability that a frame can be successfully received by the destination node without retransmission. (TIF)

S6 Fig The throughput and effective throughput of the network during the simulation. (TIF)

S7 Fig (a) The average delay changed with the number of nodes; (b) the maximum delay changed with the number of nodes. (TIF)

S8 Fig (a) The successful transmission probability of frames with the number of nodes; (b) the effective throughput network with the number of nodes. (TIF)

S9 Fig The delay of each frame sent by the four nodes. (TIF)

S10 Fig The probability that a frame can be successfully received by the destination node without retransmission. (TIF)

S11 Fig The throughput and effective throughput of the network during the simulation. (TIF)

S12 Fig The delay of each frame sent by the four nodes. (TIF)

S13 Fig The probability that a frame can be successfully received by the destination node without retransmission. (TIF)

S14 Fig The throughput and effective throughput of the network during simulation. (TIF)

S15 Fig The delay of each frame sent by the four nodes. (TIF)

S16 Fig The probability that a frame can be successfully received by the destination node without retransmission. (TIF)

S17 Fig The throughput and effective throughput of the network during the simulation. (TIF)

S18 Fig (a) The average delay changed with the network load; (b) the maximum delay changed with the network load. (TIF)

S19 Fig (a) The successful transmission probability of frames with the network load; (b) the network throughput changed with the network load. (TIF)

S1 File Appendix. (DOCX)
