# Supplementary material for: Improved MAC layer protocol of Wifi for satellite network
Source: PLoS One. 2019 Sep 6;14(9):e0221551. doi: 10.1371/journal.pone.0221551 (PMC6730845; doi:10.1371/journal.pone.0221551)
Supplement: S1 Dataset — This document contains a link to get the raw data. (DOCX) [file pone.0221551.s001.docx]

Our simulation data has been fully reflected in the manuscript, In addition, we have uploaded a package of code, original data and figures to Github "https://github.com/sili95/MAC-layer-algorithm-simulation.git". We hope that can be convenient for readers to view.

Link："https://github.com/sili95/MAC-layer-algorithm-simulation.git".
