# Supplementary figures and images for: Improved MAC layer protocol of Wifi for satellite network
Source: PLoS One. 2019 Sep 6;14(9):e0221551. doi: 10.1371/journal.pone.0221551 (PMC6730845; doi:10.1371/journal.pone.0221551)

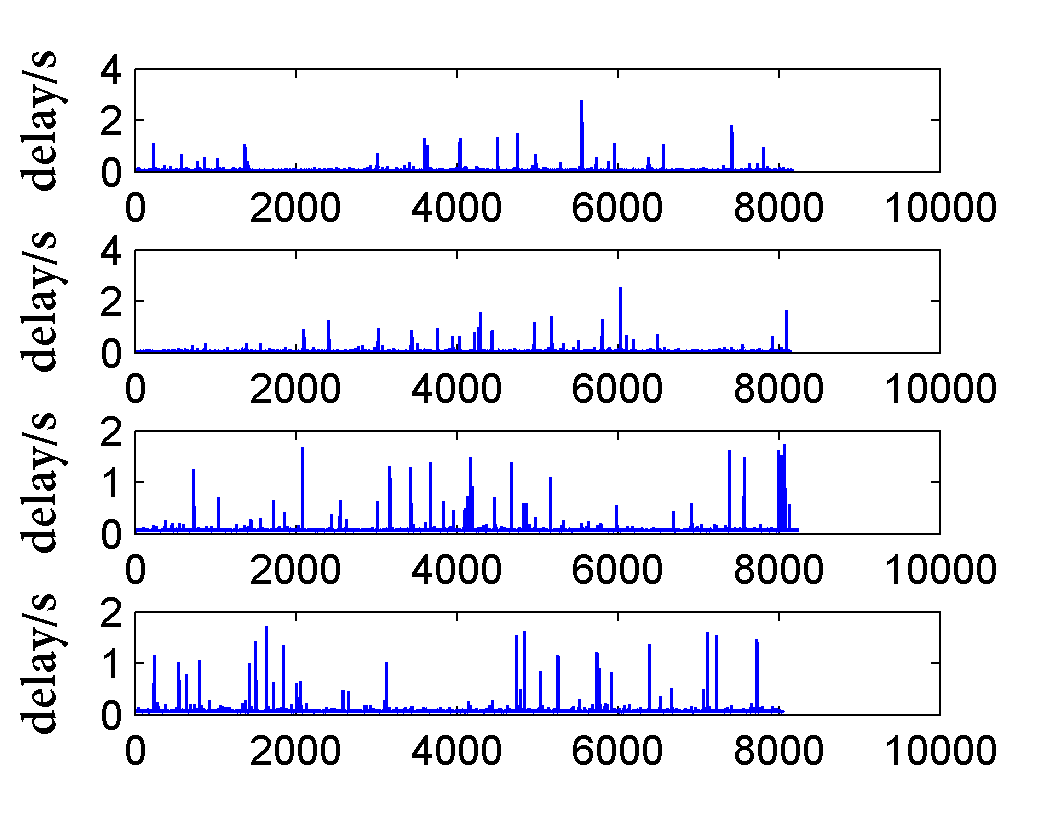

Supplement: S1 Fig — (TIF) [file pone.0221551.s002.tif]

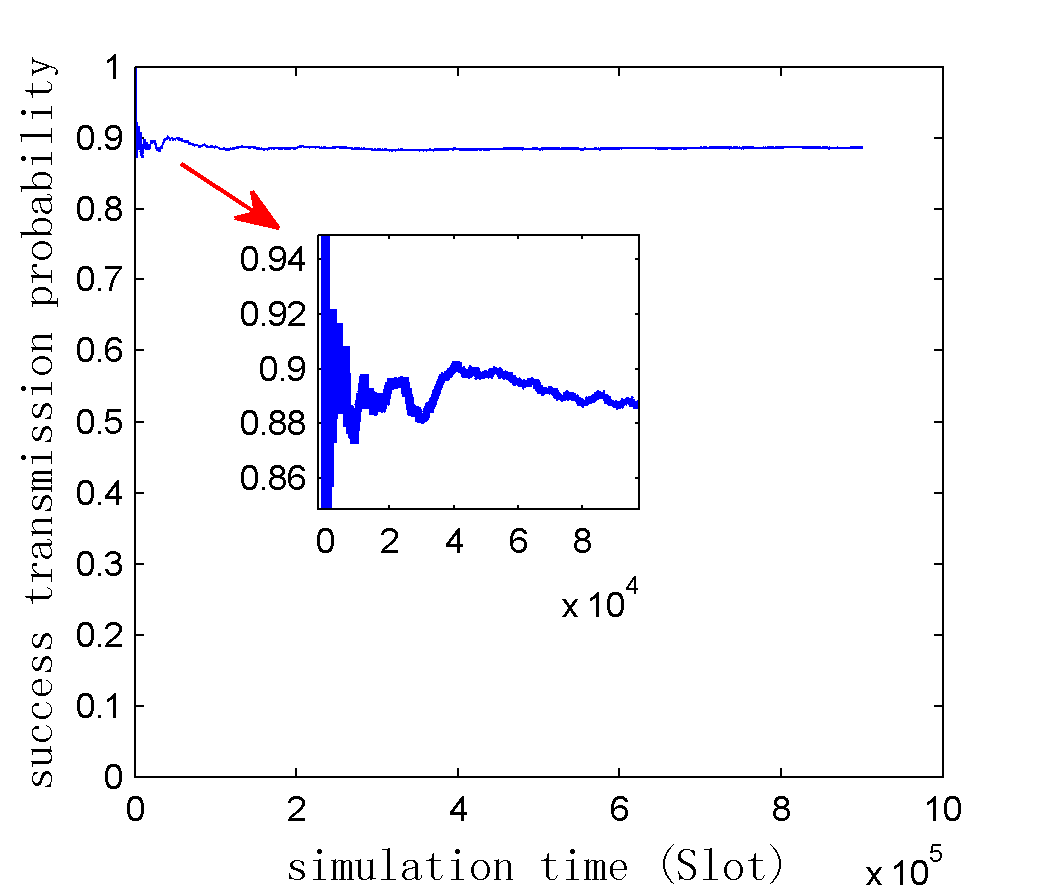

Supplement: S2 Fig — (TIF) [file pone.0221551.s003.tif]

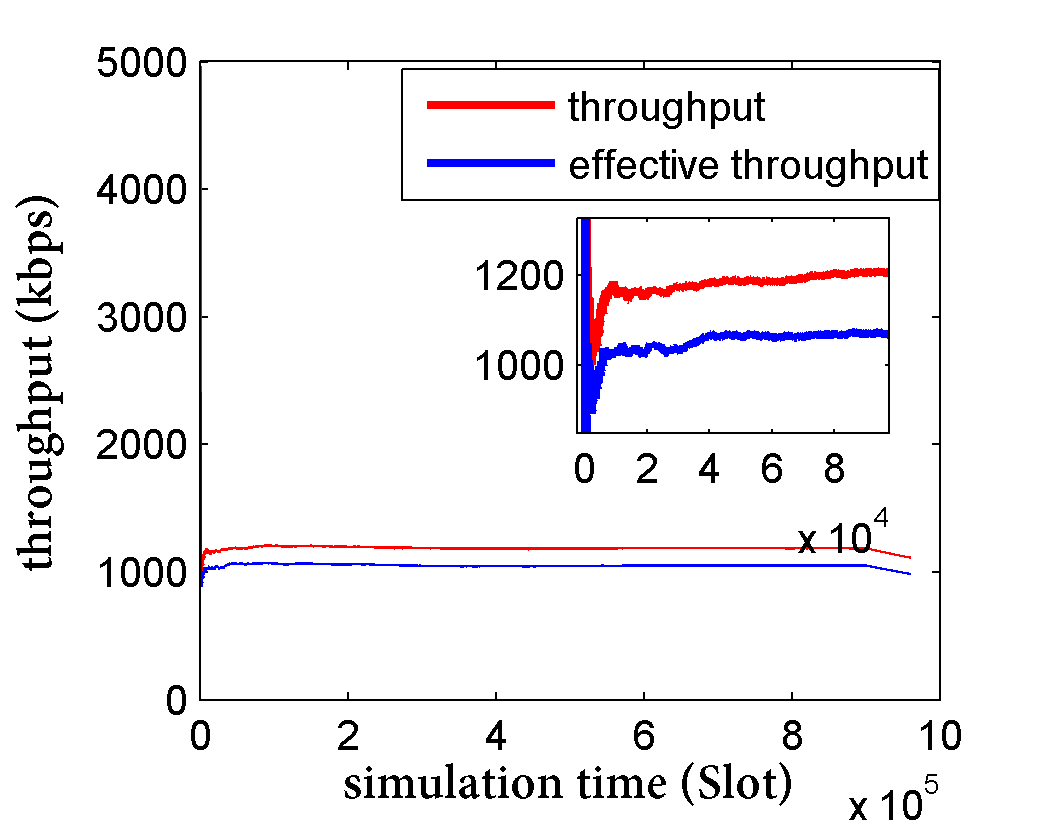

Supplement: S3 Fig — (TIF) [file pone.0221551.s004.tif]

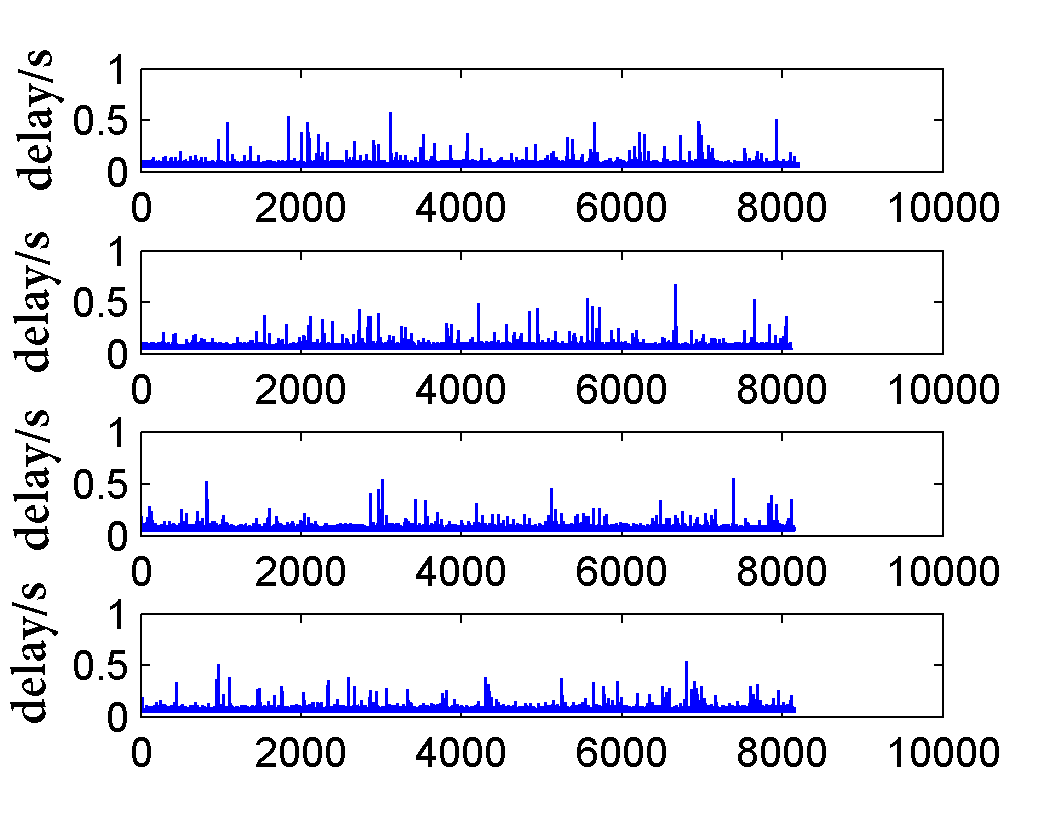

Supplement: S4 Fig — (TIF) [file pone.0221551.s005.tif]

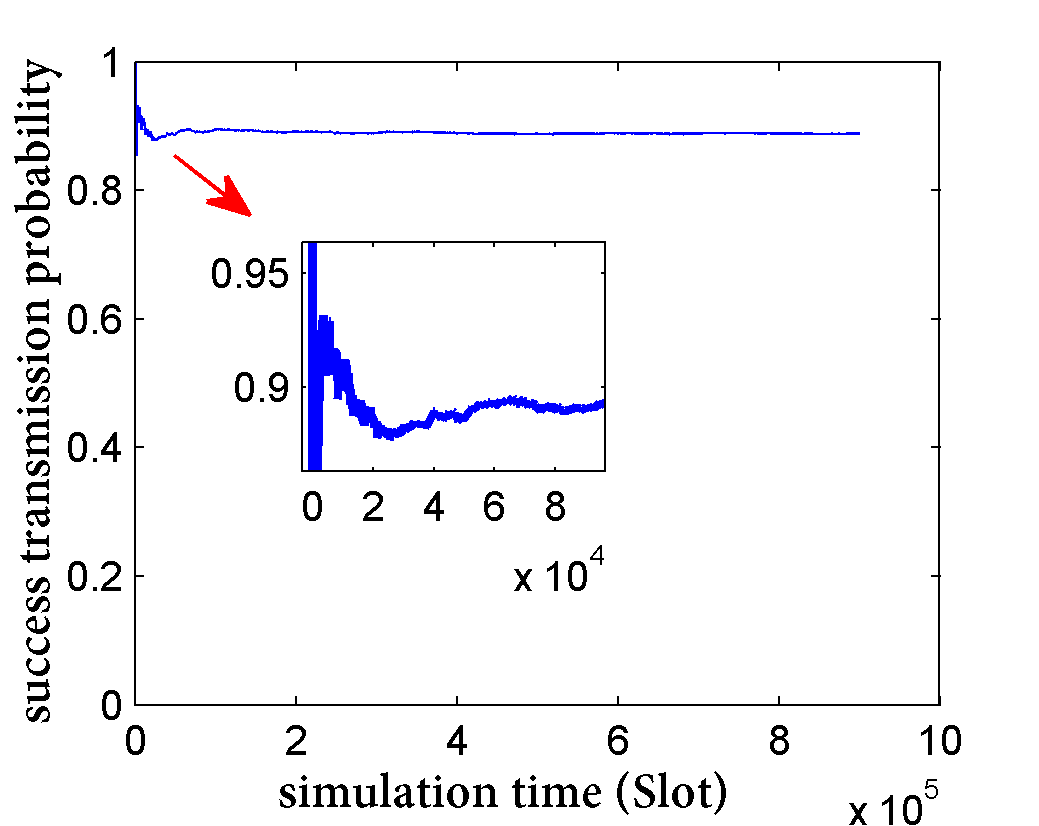

Supplement: S5 Fig — (TIF) [file pone.0221551.s006.tif]

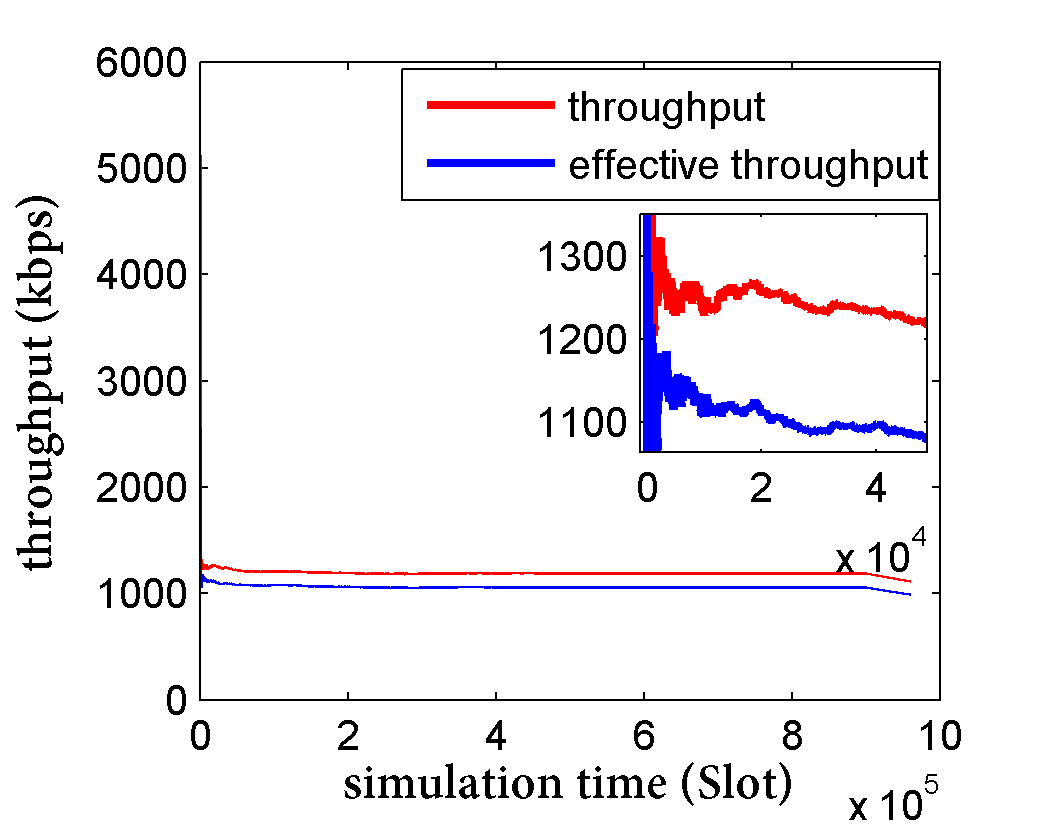

Supplement: S6 Fig — (TIF) [file pone.0221551.s007.tif]

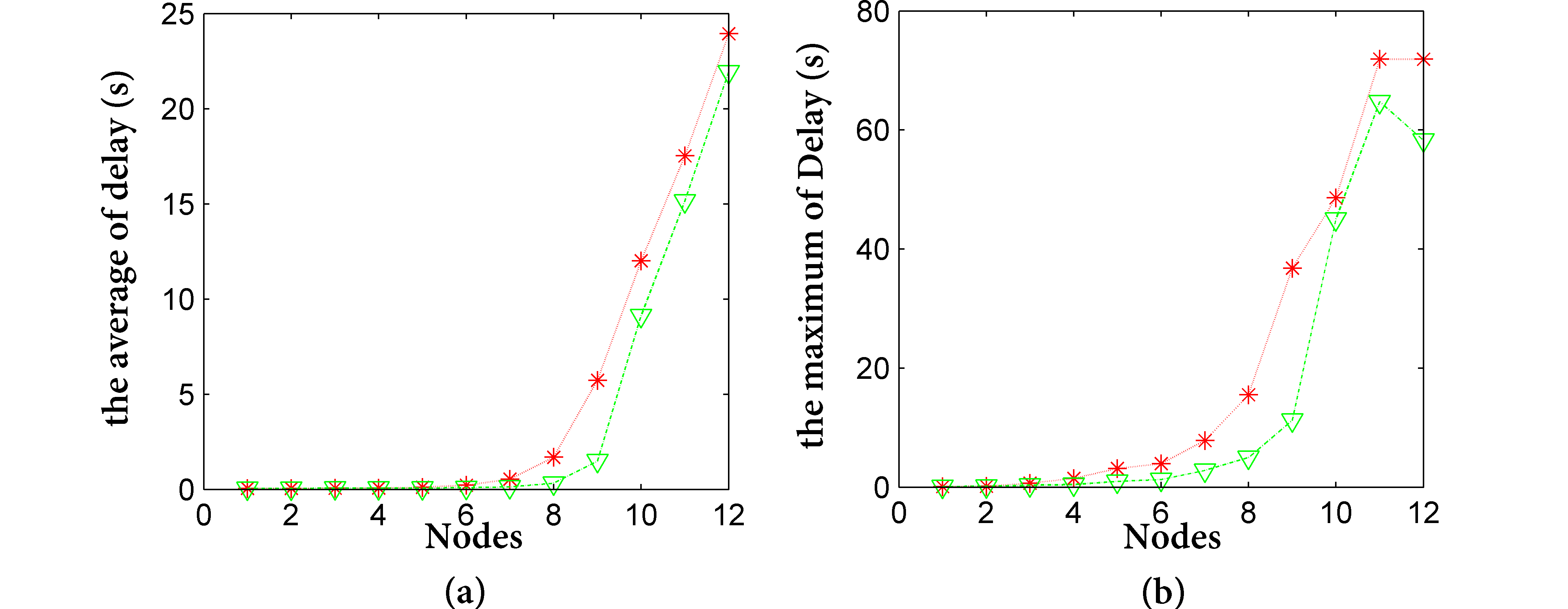

Supplement: S7 Fig — (TIF) [file pone.0221551.s008.tif]

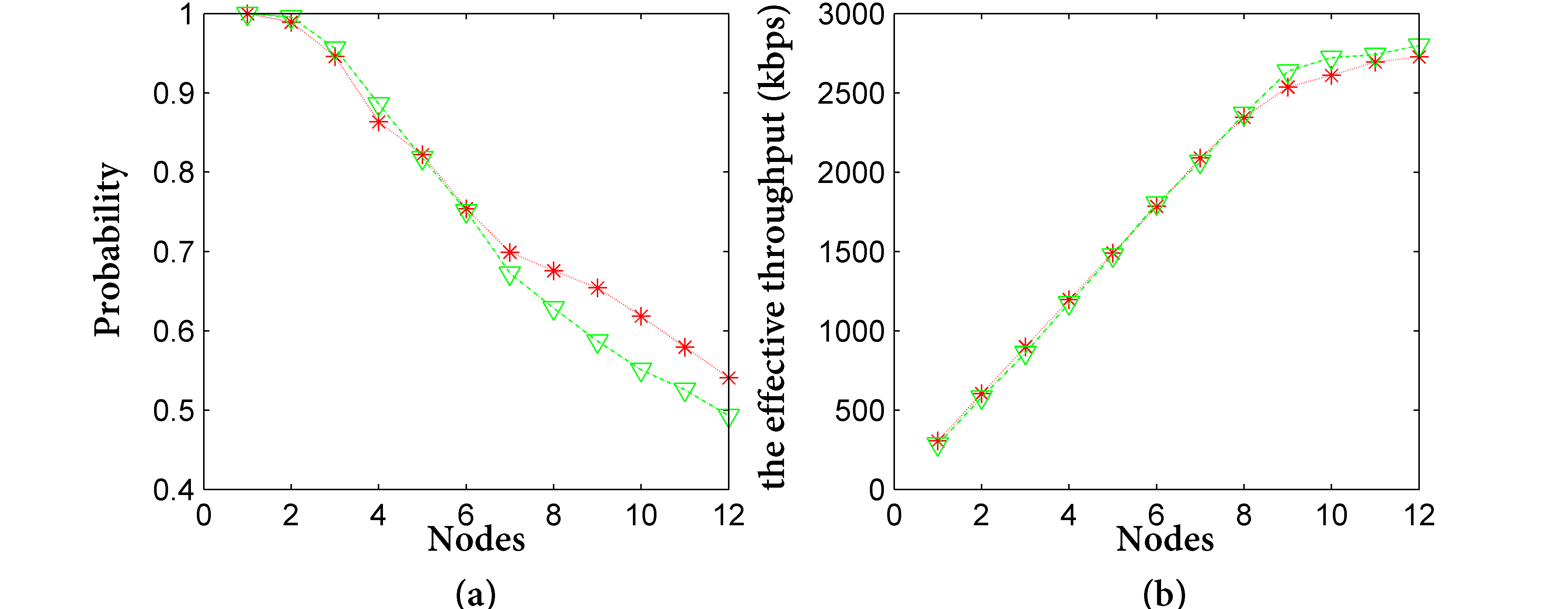

Supplement: S8 Fig — (TIF) [file pone.0221551.s009.tif]

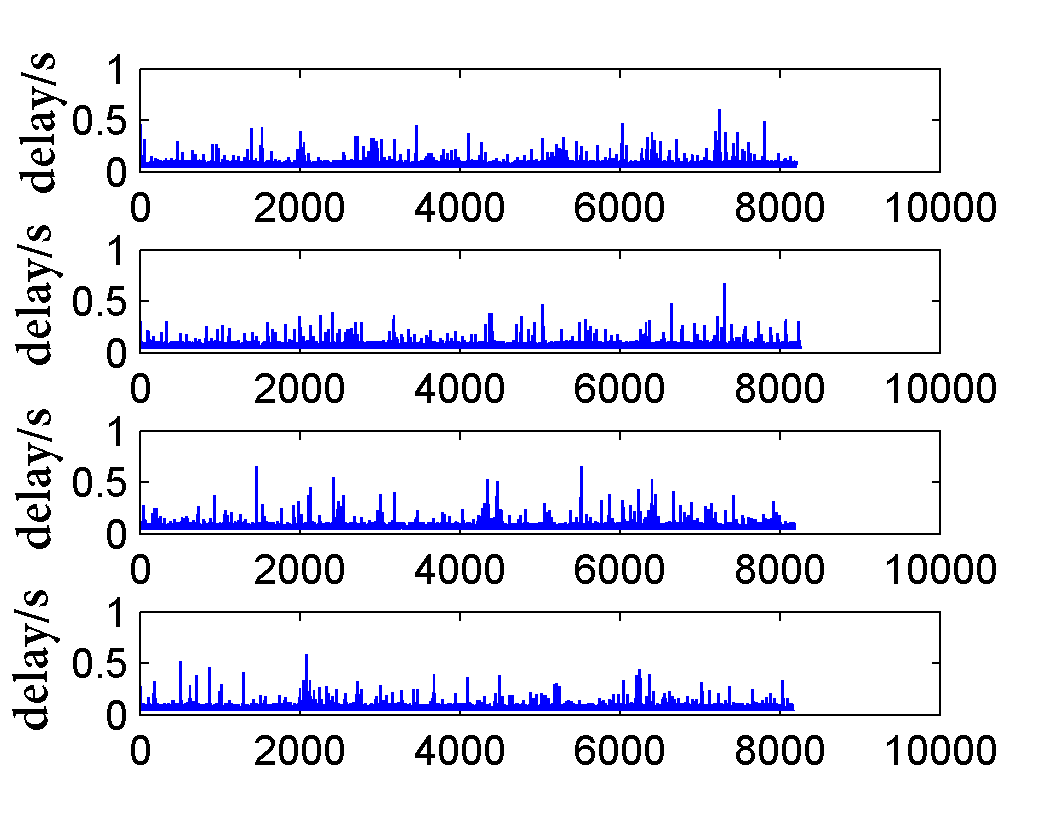

Supplement: S9 Fig — (TIF) [file pone.0221551.s010.tif]

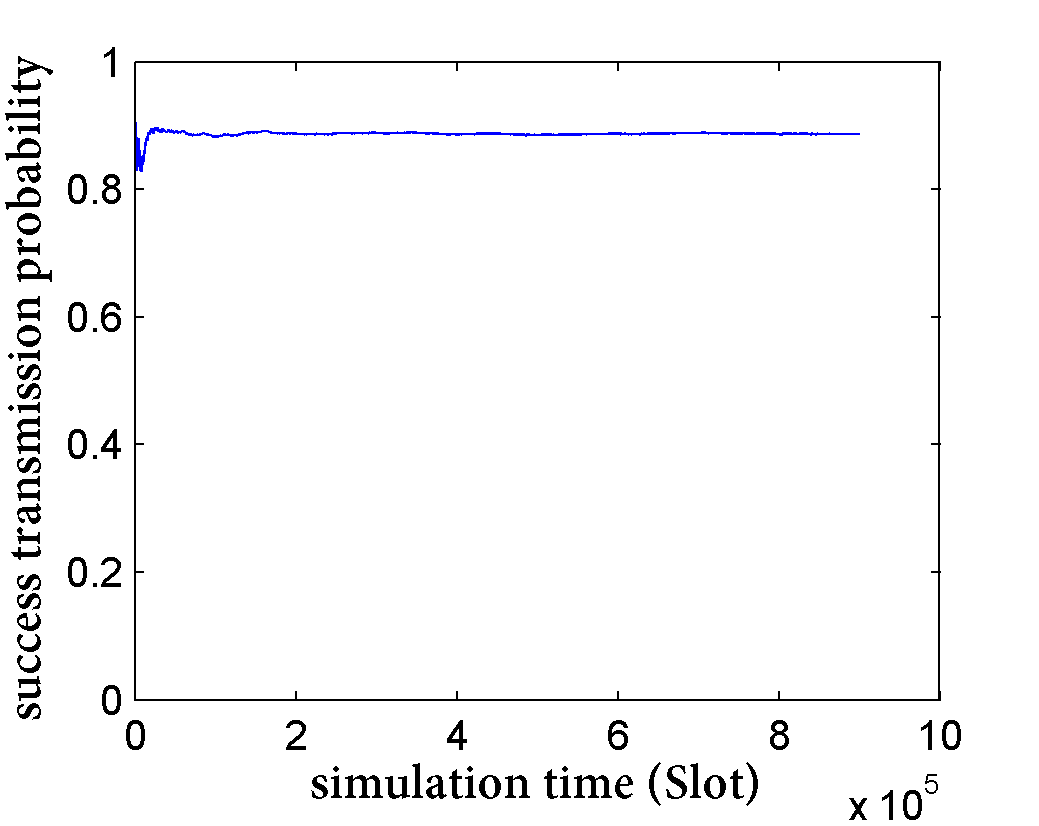

Supplement: S10 Fig — (TIF) [file pone.0221551.s011.tif]

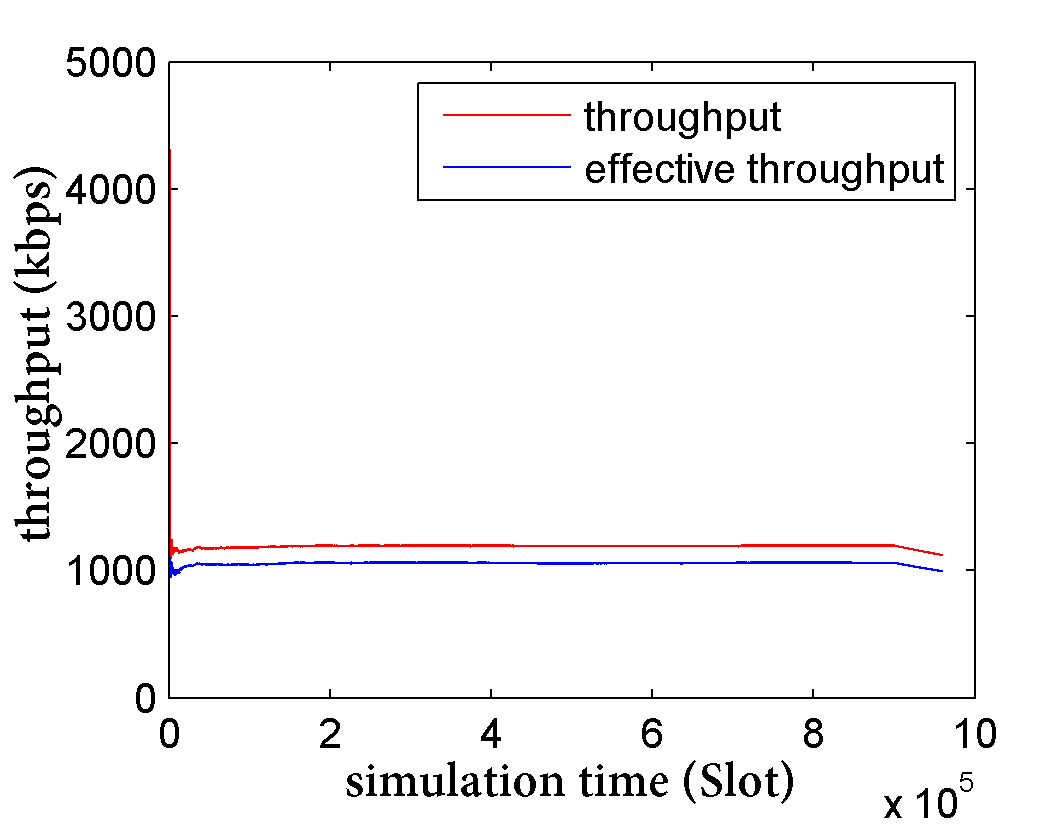

Supplement: S11 Fig — (TIF) [file pone.0221551.s012.tif]

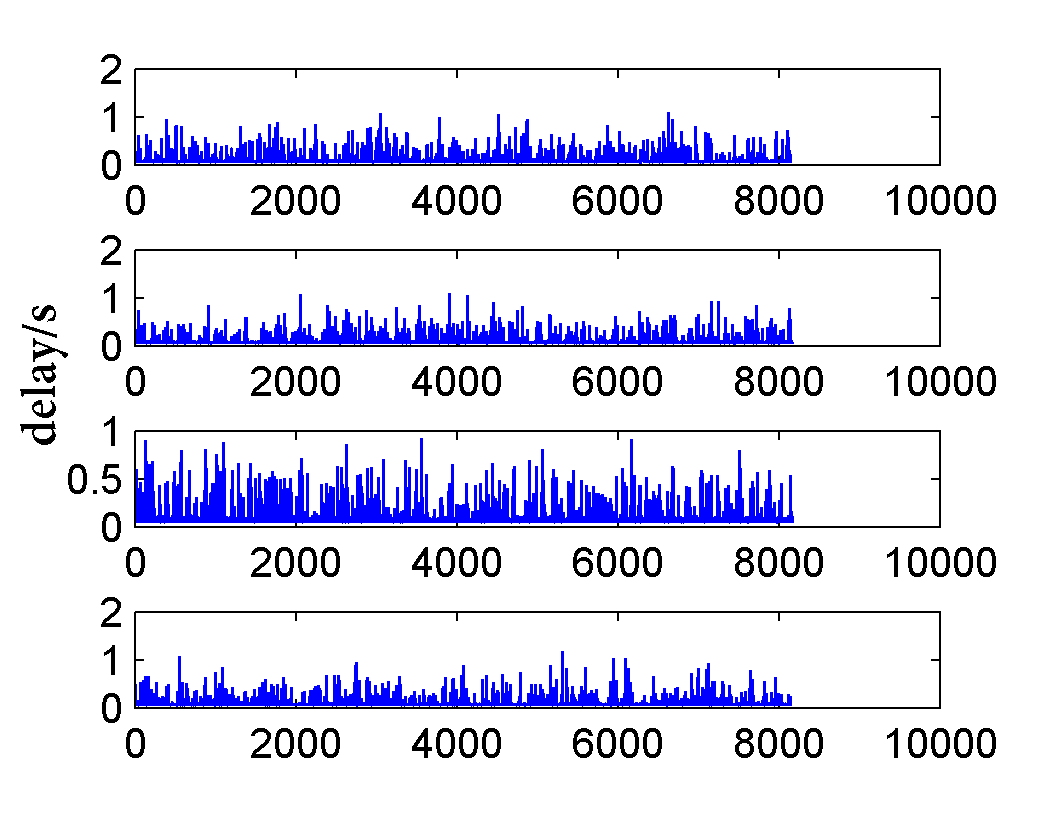

Supplement: S12 Fig — (TIF) [file pone.0221551.s013.tif]

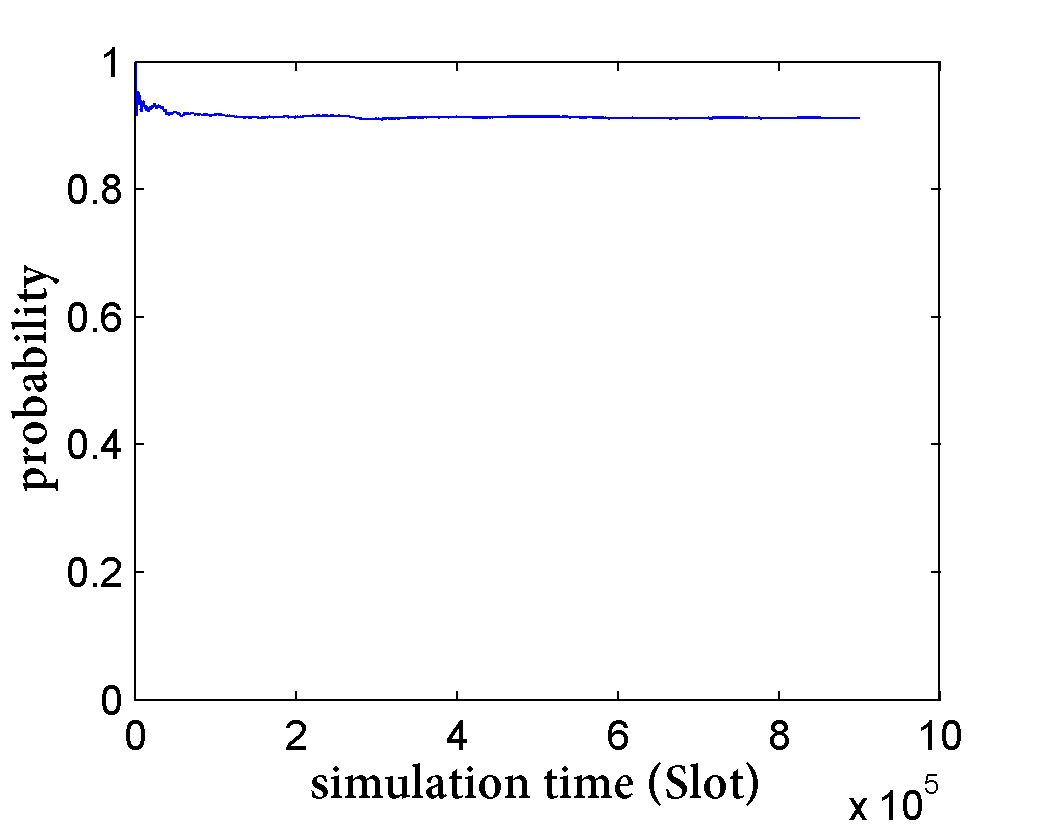

Supplement: S13 Fig — (TIF) [file pone.0221551.s014.tif]

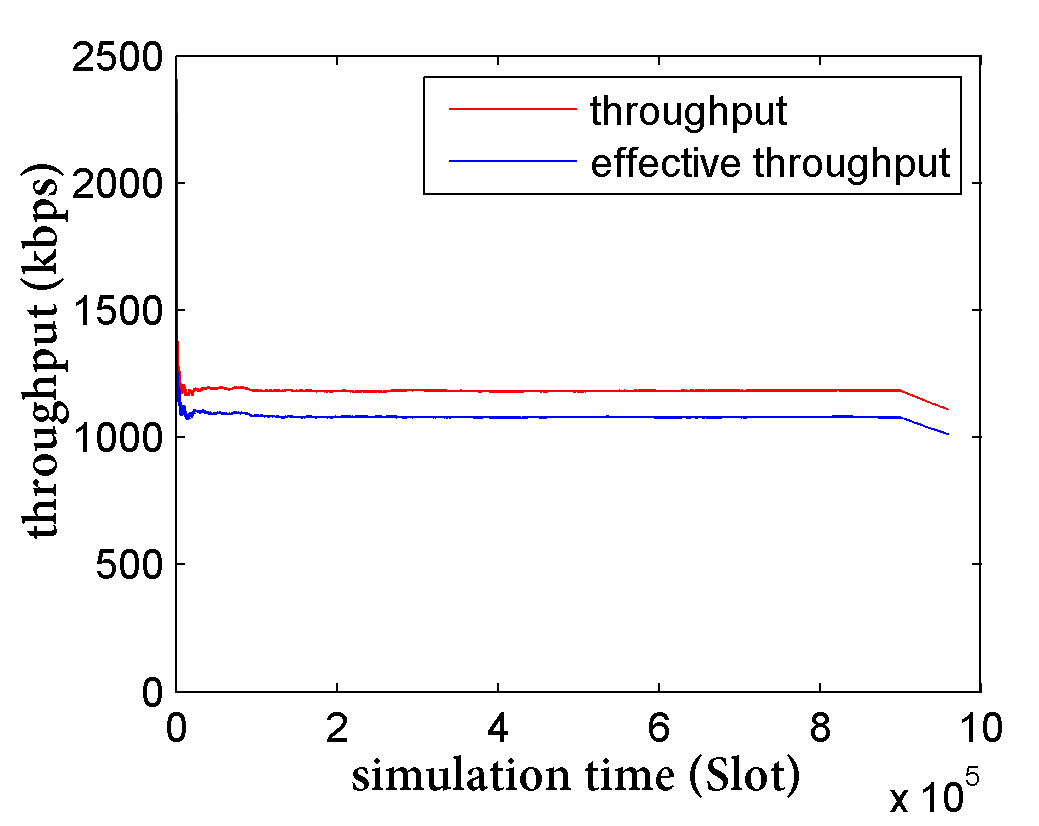

Supplement: S14 Fig — (TIF) [file pone.0221551.s015.tif]

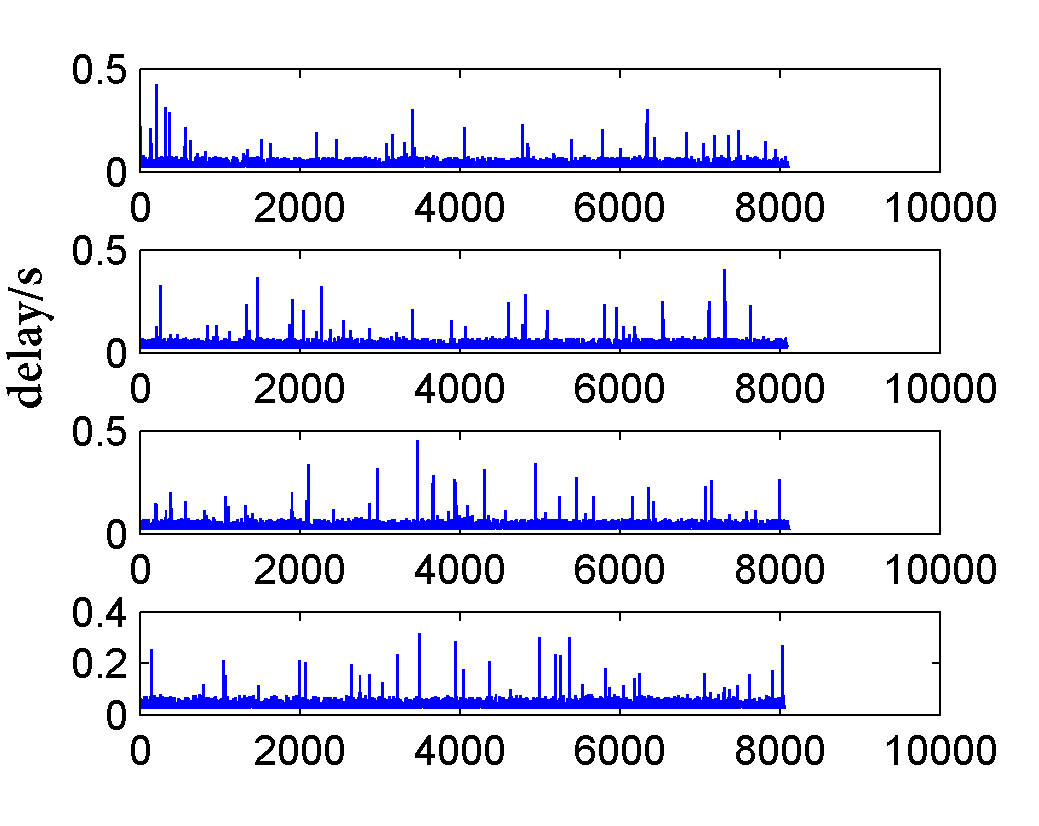

Supplement: S15 Fig — (TIF) [file pone.0221551.s016.tif]

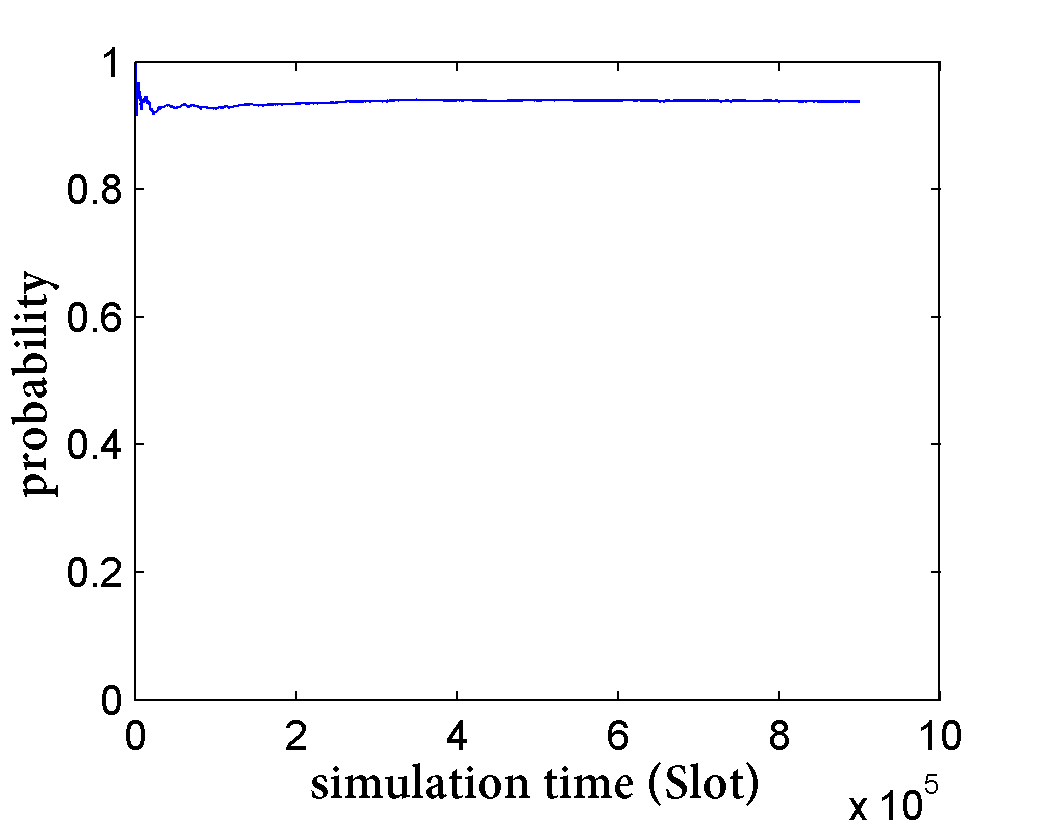

Supplement: S16 Fig — (TIF) [file pone.0221551.s017.tif]

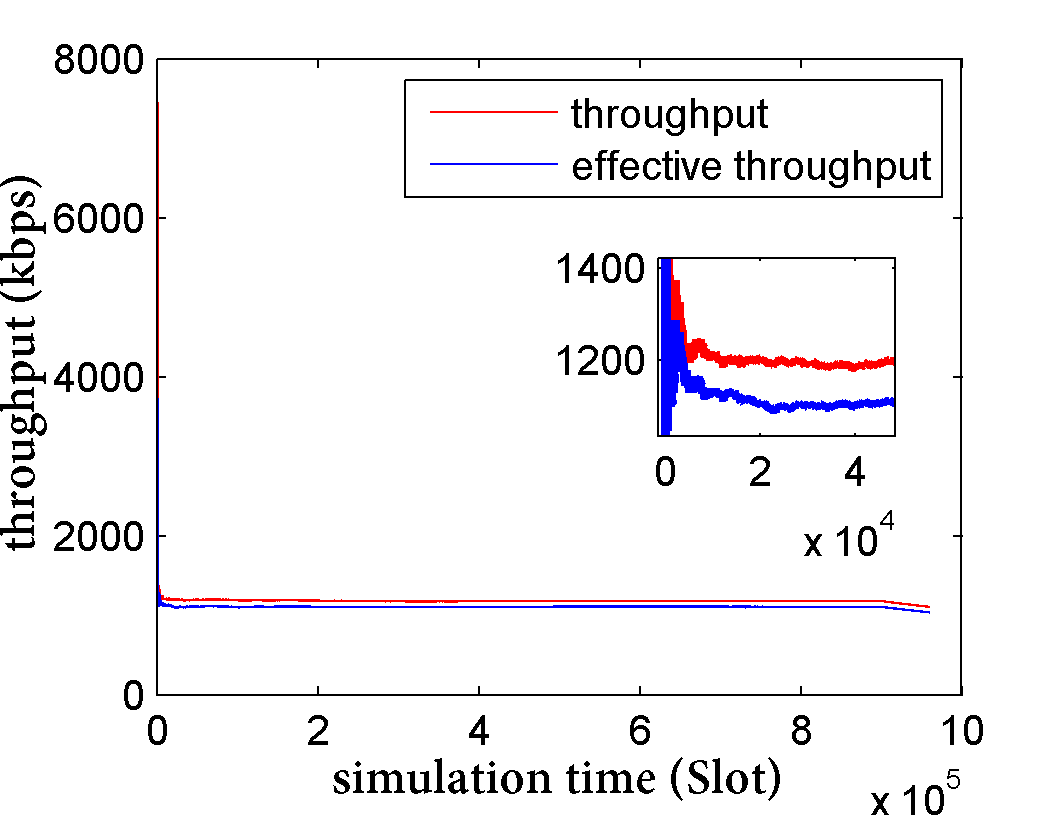

Supplement: S17 Fig — (TIF) [file pone.0221551.s018.tif]

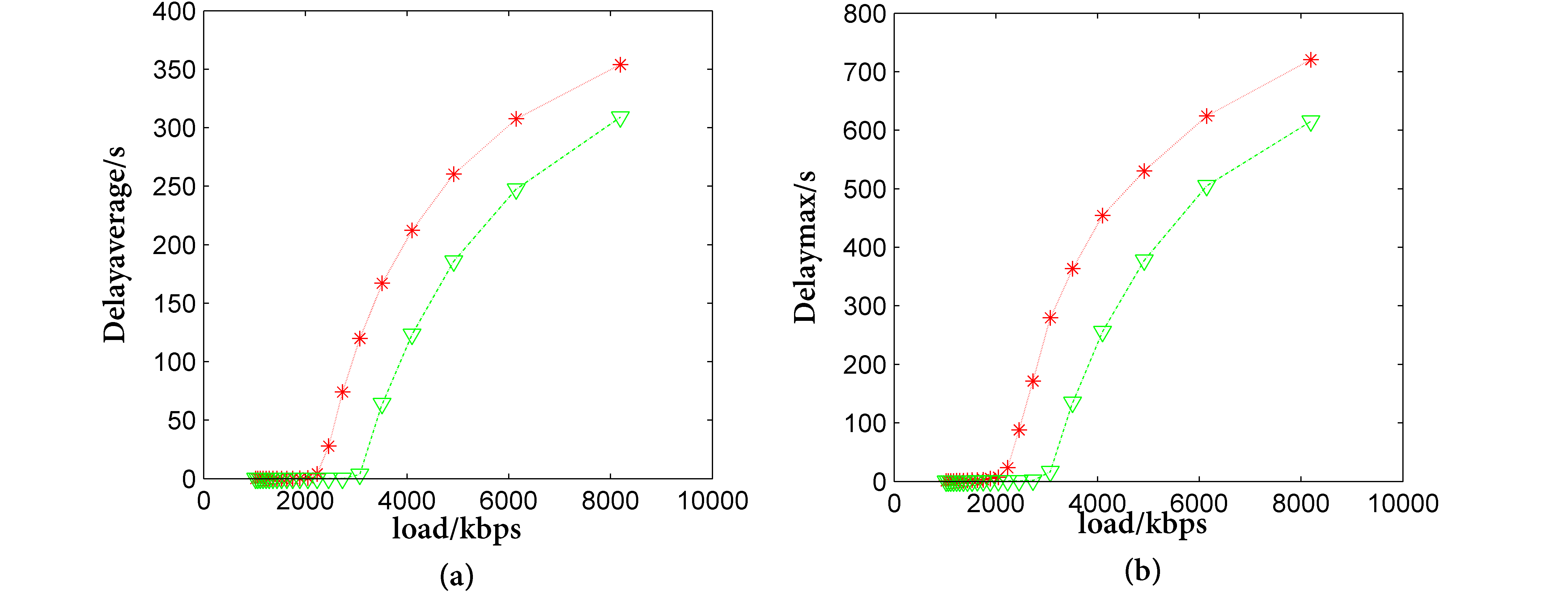

Supplement: S18 Fig — (TIF) [file pone.0221551.s019.tif]

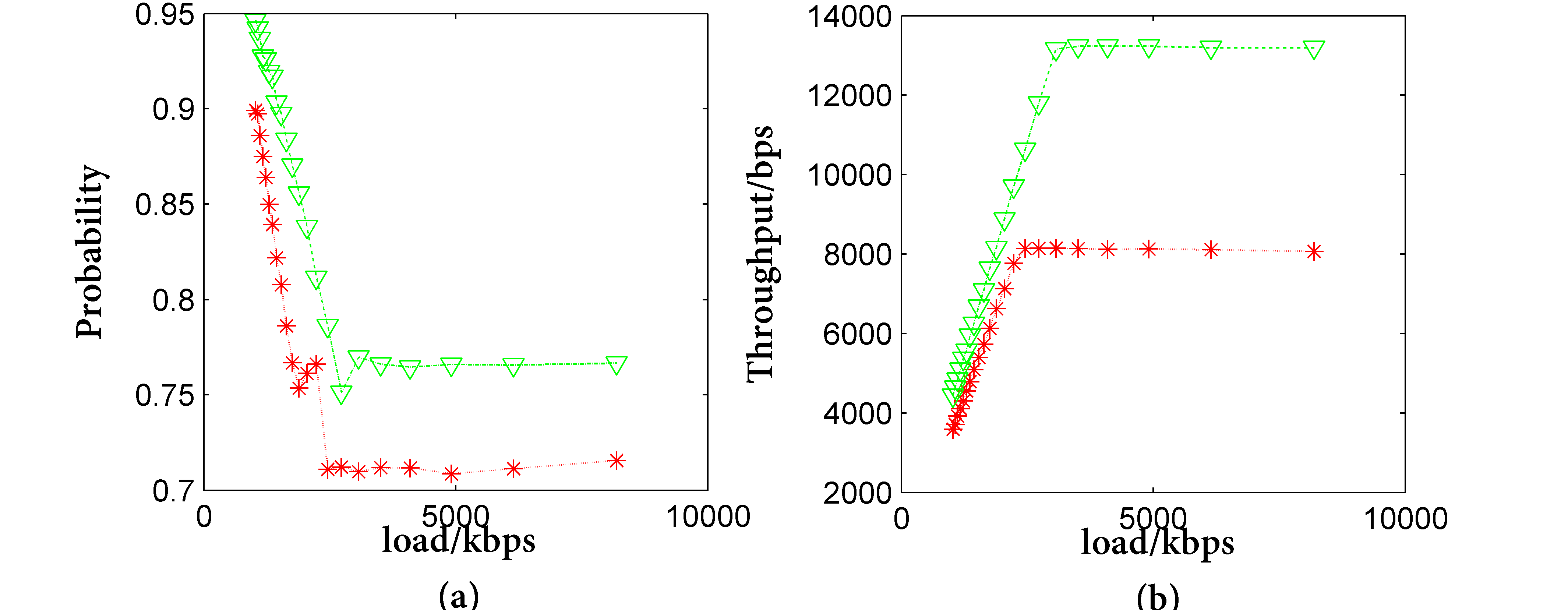

Supplement: S19 Fig — (TIF) [file pone.0221551.s020.tif]
