# Supplementary material for: Improved MAC layer protocol of Wifi for satellite network
Source: PLoS One. 2019 Sep 6;14(9):e0221551. doi: 10.1371/journal.pone.0221551 (PMC6730845; doi:10.1371/journal.pone.0221551)
Supplement: S1 File — (DOCX) [file pone.0221551.s021.docx]

Appendix A

The timing model of 802.11 is shown in Fig 1 [1].

**Fig 1.** The time series model of 802.11.

As shown in Fig 1, the back-off timer of satellite node-1 is first rolled back to 0. The RTS frame is first sent to satellite node-2. After the satellite node-2 successfully receives the RTS frame, it waits for the SIFS to broadcast a CTS frame to all nodes. All nodes of the network will receive a CTS frame containing the duration of this communication required. During this duration time, other satellite nodes will not attempt to access the media, and will suspend their random back-off timer, and the back-off timer count will not continue to count down.

When satellite node-1 receives the CTS, it finds that the frame is the feedback of the previous RTS. At this time, the satellite node-1 knows that the media is idle. After waiting for the SIFS, the satellite node-1 sends a data frame, and the satellite node-2 feeds back an ACK to the satellite node-1, thereby completing one transmission.

Traditional WiFi protocol can be used in many specific scenarios by improving network performance, which can support software definition, support the mobility of nodes, achieve load balancing [2-5]. We can also increase the throughput through frequency division multiplexing and achieve anti-interference through Measurement Based Self Organization [6, 7]. In terms of Qos quality assurance, channel design routing can be allocated based on dynamic perception of channels to improve network performance [8, 9].In this paper, the BEB algorithm of WiFi protocol is improved to adapt to the transmission requirements of inter-satellite network.

In CSMA/CA, the node needs to perform the back-off collision avoidance process before each transmission. During the avoidance process, the node will listen in every time slot. If the media listening result is idle, the back-off timer count is decremented by 1. If the media is busy, the back-off timer is suspended, and the node can send data only when the timer is 0. Combining the time characteristics of the space network, in the process of back-off collision avoidance, when the number of collision times is 1 to 8 times, the size of the competition window doubles, and when it is greater than 8 times, the window size remains unchanged. When it is greater than 12 times, the packet loss processing is performed and the transmission fails. It can be seen that in the traditional 802.11 back-off algorithm, the contention window has fixed maximum and minimum values, and CWmax and CWmin applicable to the space satellite network can be found in the simulation process. In the simulation, we choose 8 slots as CWmin and 256 slots as CWmax. During the back-off collision avoidance process, the law of the back-off timer window increasing with the number of collision times is as shown in Fig 2.

**Fig 2.** The size of the competition window varies with the number of collisions.

The unit of the ordinate in the Fig 2 is CWmin, which represents the value of the minimum competition window. And the back-off time is calculated as (1).

$$back-off=Integer((CW\text{-1)}\times RN)$$

(1)

Where RN is pseudo-Random Number in the range 0.0 to 1.0. Integer indicates that the result of the calculation is rounded up to the nearest integer.

References

1. IEEE Standard for Wireless LAN Medium Access Control (MAC) and Physical Layer (PHY) specifications," in IEEE Std 802.11. p.1-445, 18 Nov. 1997. doi: 10.1109/IEEESTD.1997.85951.
2. Singh KVK and Pandey M, "Software-defined mobility in IP based WiFi networks: Design proposal and future directions," 2016 IEEE International Conference on Advanced Networks and Telecommunications Systems (ANTS), Bangalore, 2016, p. 1-6.
3. Fontes RR, Afzal S, Brito SHB, Santos MAS and Rothenberg CE, "Mininet-WiFi: Emulating software-defined wireless networks," 2015 11th International Conference on Network and Service Management (CNSM), Barcelona, 2015, p. 384-389.
4. Chen Z, Manzoor S, Gao YY and Hei XJ, "Achieving Load Balancing in High-Density Software Defined WiFi Networks," 2017 International Conference on Frontiers of Information Technology (FIT), Islamabad, 2017, p. 206-211.
5. Gao YY, Dai L and Hei XJ, "Throughput Optimization of Multi-BSS IEEE 802.11 Networks With Universal Frequency Reuse," in IEEE Transactions on Communications, vol. 65, no. 8, p. 3399-3414, Aug. 2017.
6. Kauffmann B, Baccelli F, Chaintreau A, Mhatre V, Papagiannaki K and Diot C, "Measurement-Based Self Organization of Interfering 802.11 Wireless Access Networks," IEEE INFOCOM 2007 - 26th IEEE International Conference on Computer Communications, Barcelona, 2007, p. 1451-1459..
7. Chakraborty S, Saha B, Bandyopadhyay S, "Dynamic channel allocation in IEEE 802.11 networks", Int. J. Comput. Appl., vol. 149, no. 1, p. 36-38, Sep. 2016.
8. Ghahfarokhi BS, "Distributed QoE-aware channel assignment algorithms for IEEE 802.11 WLANs", Wireless Netw., vol. 21, no. 1, p. 21-34, 2015.
9. IEEE, 802.11ac-2013, "IEEE Standard for Information Technology–Telecommunications and Information Exchange Between Systems—Local and Metropolitan Area Networks—Specific Requirements—Part 11: Wireless LAN Medium Access Control (MAC) and Physical Layer (PHY) Specifications—Amendment 4: Enhancements for Very High Throughput for Operation in Bands Below 6 GHz", Dec. 2013. Valuable from: <https://standards.ieee.org/standard/802_11ac-2013.html>.
